# Spontaneous pregnancy-associated coronary artery dissection: a case report on diagnostic and therapeutic challenges

**DOI:** 10.1093/ehjcr/ytae204

**Published:** 2024-04-20

**Authors:** Ailís Ceara Haney, Deborah Siry, Isabel Amber-Rose Hoerbrand, Philipp Ehlermann, Jan Beckendorf

**Affiliations:** Department of Internal Medicine III, Division of Cardiology, University Hospital Heidelberg, Im Neuenheimer Feld 410, 69120 Heidelberg, Germany; Department of Internal Medicine III, Division of Cardiology, University Hospital Heidelberg, Im Neuenheimer Feld 410, 69120 Heidelberg, Germany; Department of Internal Medicine III, Division of Cardiology, University Hospital Heidelberg, Im Neuenheimer Feld 410, 69120 Heidelberg, Germany; Department of Internal Medicine III, Division of Cardiology, University Hospital Heidelberg, Im Neuenheimer Feld 410, 69120 Heidelberg, Germany; Department of Internal Medicine III, Division of Cardiology, University Hospital Heidelberg, Im Neuenheimer Feld 410, 69120 Heidelberg, Germany

**Keywords:** Pregnancy, Spontaneous coronary artery dissection, Pregnancy heart team, Intravascular imaging, Case report

## Abstract

**Background:**

One of the main causes of myocardial infarction during pregnancy is spontaneous coronary artery dissection. This is ascribed to hormonal changes during pregnancy leading to a weakening of the vessel wall and haemodynamic changes especially during childbirth. Management options include conservative medical treatment and percutaneous coronary intervention, depending on clinical presentation.

**Case summary:**

A 37-year-old woman presented with typical chest pain six weeks after giving birth to her third child. Echocardiography revealed a moderate reduction in systolic function. Initial invasive coronary angiography showed no abnormalities. After cardiac magnetic resonance demonstrated extensive scar, invasive coronary angiography was repeated including intravascular imaging. A dissection of the left anterior descending artery was visualized and treated by percutaneous coronary intervention and stenting. Left ventricular function was normalized at three-month follow-up. In this educational case report, we highlight the diagnostic and therapeutic challenges when treating this special patient cohort and the importance of cardiovascular imaging.

**Discussion:**

Pregnancy-associated spontaneous coronary dissection is a potential differential diagnosis when treating post-partum women with recent onset chest pain. Management is challenging and intravascular imaging to visualize dissection should be performed during invasive coronary angiography. Patients require interdisciplinary care within a pregnancy heart team.

**Learning points**

- Management of pregnancy-associated spontaneous coronary artery dissection (SCAD) may be challenging, especially concerning decision on revascularization. Conservative management is the preferred treatment option in stable patients with SCAD.
- Intravascular imaging may provide crucial information to guide the decision-making process.
- Interdisciplinary care in the pregnancy heart team is essential when treating patients during pregnancy or the post-partum period.

## Introduction

Cardiac disease should be taken into consideration within a patient population of pregnant women presenting with chest pain as it accounts for >20% of all maternal cardiac deaths. The most common cause for peripartum acute myocardial infarction (AMI) is spontaneous coronary artery dissection (SCAD), most commonly occurring at the endstages of pregnancy or during early post-partum.

In this article, we present a case of a 37-year-old patient who presented with unstable angina six weeks post-partum and was diagnosed with pregnancy-associated spontaneous coronary artery dissection (P-SCAD) during hospitalization with the help of intravascular imaging.

## Summary figure

**Figure:**
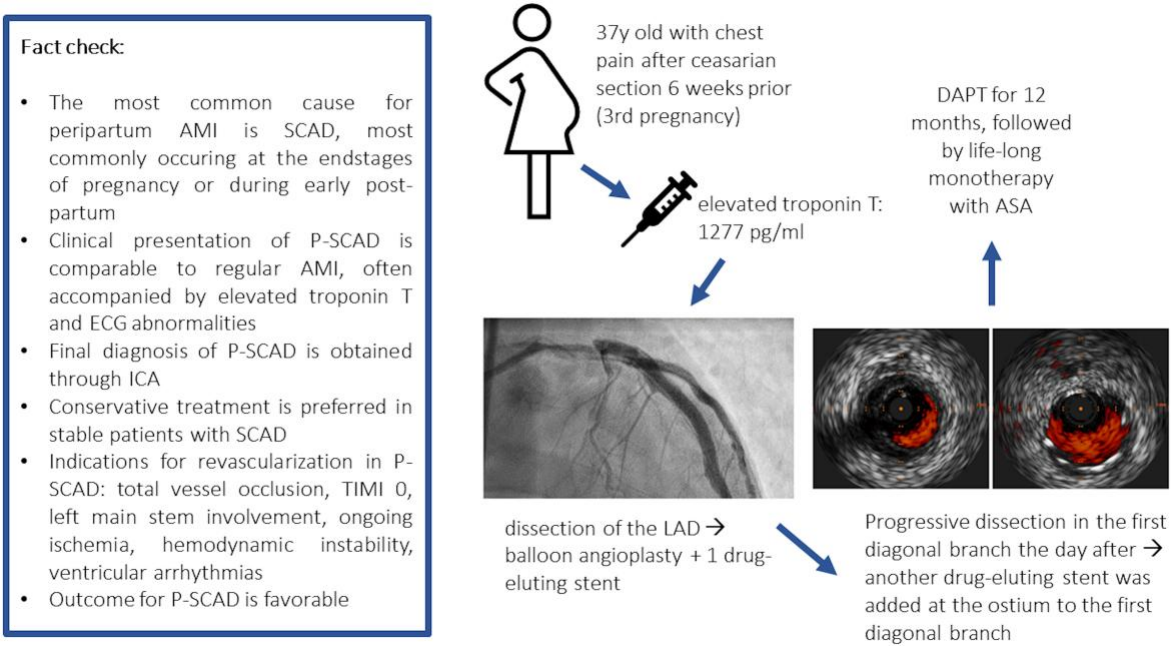


## Case summary

A 37-year-old woman presented to the emergency department, reporting constant chest pain radiating to her left arm that had started 10 days prior to presentation and had exacerbated the day before (grade IV according to the Canadian Cardiovascular Society Angina Score). Caesarean section had been performed six weeks prior to presentation after her third pregnancy (gravida 3 para 3). She had not experienced any complications during her pregnancy, notably no elevated blood pressure. Likewise, the delivery by planned caesarean section was performed without complications. History included thalassaemia minor. She did not take any regular medication, had no allergies and no cardiovascular risk factors. Her two previous pregnancies had been uneventful.

At presentation vital parameters were predominantly within normal range (blood pressure 123/78 mmHg, heart rate 102/min, temperature 37.4°C). Physical examination was unremarkable. The electrocardiogram (ECG) showed no abnormalities. High-sensitivity troponin T at presentation was significantly elevated with 1277 pg/mL and remained constantly high after 90 min: 1245 pg/mL. NT-pro-BNP was also increased: 3310 ng/L.

**Figure 1:**
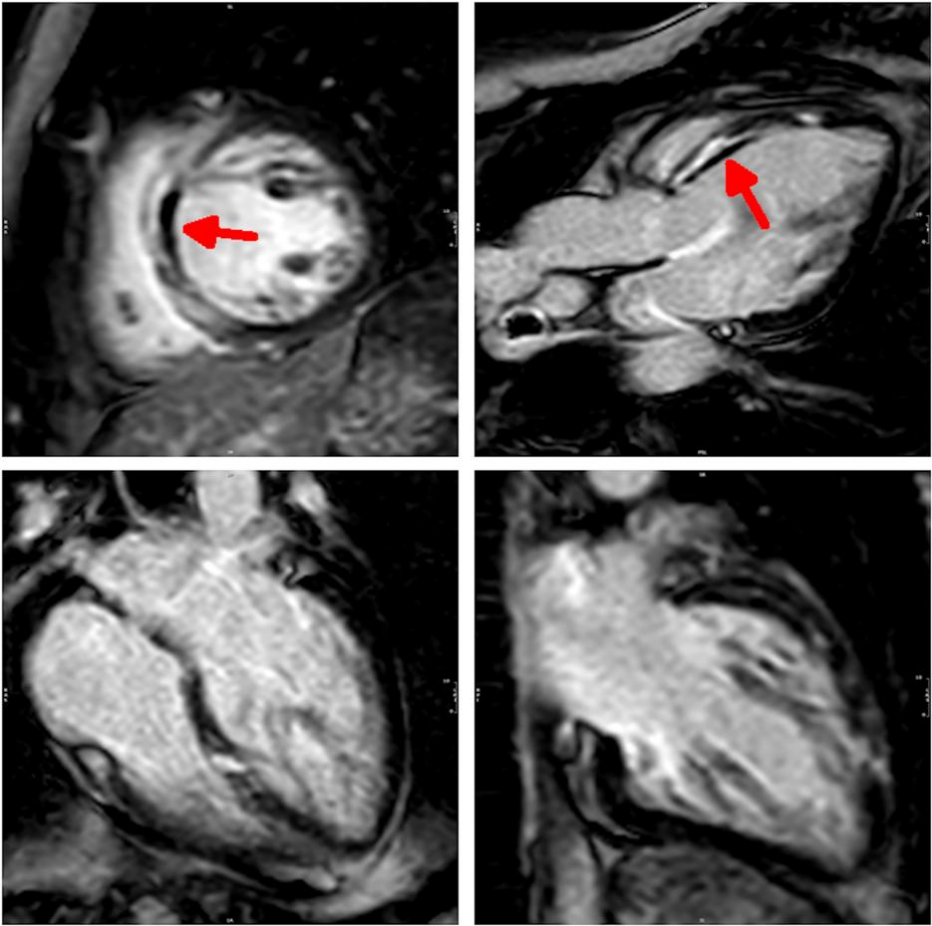
Late gadolinium enhancement cardiac MR 3 days after initial presentation. Infarct-like LGE is visualized in septal and anterior segments on short axis, three-chamber, four-chamber, and two-chamber views, including microvascular obstruction.

**Figure 2:**
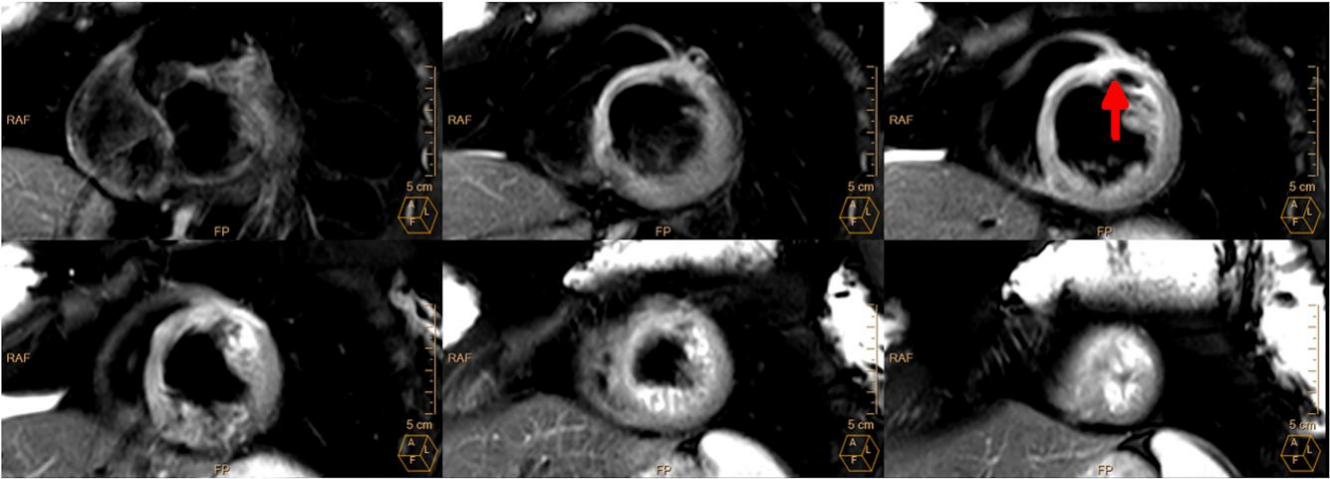
Acute myocardial oedema on cardiac magnetic resonance 3 days after initial presentation. T2-weighted short axis CMR imaging shows acute myocardial oedema in septal and anterior segments, corresponding with LGE imaging.

The patient was directly referred to our catheter laboratory. However, invasive coronary angiography (ICA) showed neither a significant coronary artery stenosis nor a coronary artery dissection (TIMI III flow). Echocardiography showed a dilated left ventricle with moderately reduced systolic function accompanied by anteroseptal and septal hypokinesia. There were no signs of right ventricular dysfunction, pulmonary embolism was therefore ruled out. Cardiac magnetic resonance (CMR) demonstrated reduced left ventricular ejection fraction (LVEF 37%) and extensive transmural late gadolinium enhancement in septal and apical territories, accompanied by oedema and microvascular obstruction, suggestive of myocardial infarction caused by thromboembolism or dissection (see *Figures 1* and *2*, see Supplementary material online, *Video 1*). A further angiological examination including colour-duplex sonography as well as a transoesophageal echocardiography was normal, showing no signs of a thrombo-embolic cause. Sarcoidosis seemed improbable after negative laboratory testing [ACE (21.1 U/L; ref.-range: 17.5–65.7 U/L), S-IL-2R (342 U/mL; ref.-range: <900/mL), calcium (2.57 mmol/L; ref.-range: 2.11–2.59 mmol/L)].

**Figure 3:**
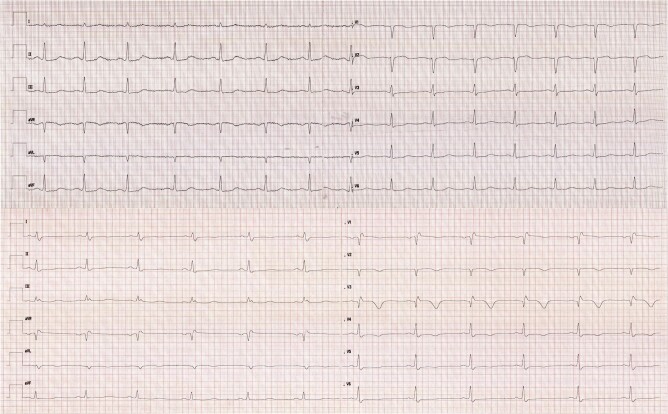
ECG at presentation (above) showing no significant ischaemic changes compared to ECG 4 days after presentation (below) demonstrating new discordant inversion of T-waves in leads V3–V6.

**Figure 4:**
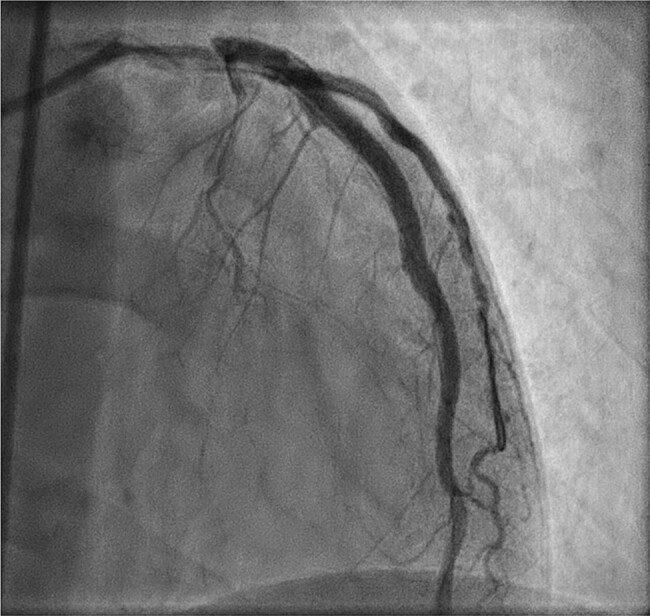
Acute coronary artery dissection of the left anterior descending artery. Coronary angiography showing dissection of the LAD, causing acute coronary syndrome. Notching is visible in the proximal LAD.

**Figure 5:**
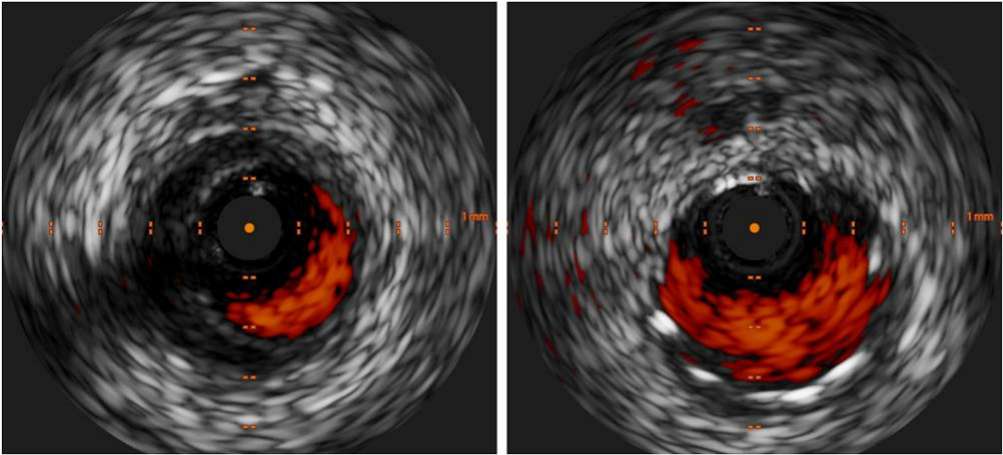
Intravascular ultrasound visualizing acute coronary artery dissection. IVUS diagnosed dissection of LAD (left) and was performed after successful stenting of dissection (right).

Four days after admission and following CMR, new discordant negative T-waves appeared on ECG in leads V3–V6 (see *Figure 3*). The patient’s chest pain persisted despite maximum antianginal therapy (bisoprolol 2.5 mg b.i.d., ISDN 20 mg b.i.d., amlodipine 5 mg o.d.). We interpreted these changes as ongoing ischaemia. After discussion of the case in our pregnancy heart team, it was decided to repeat ICA including intravascular imaging as the risk of occlusion of the proximal LAD with potentially severe outcomes was deemed higher than the risk of percutaneous coronary intervention (PCI). A dissection of the left anterior descending artery was visualized and subsequently treated by high-pressure balloon angioplasty and one drug-eluting stent guided by intravascular ultrasound (IVUS) in order to optimize stent strut apposition (see *Figures 4* and *5*, see Supplementary material online, *Videos S2*–*S5*). TIMI III flow was achieved (see Supplementary material online, *Video 6*). One day later, the patient reported left thoracic pain once again now accompanied by ST-segment elevations in aVR and V1. Emergency ICA was initiated that showed a progressive dissection into the first diagonal branch of the left anterior descending artery (LAD). T-stenting of the first diagonal branch was performed using a drug-eluting stent. The patient was subsequently asymptomatic, and the ECG changes receded. Further hospital stay remained without any complications and the patient was discharged within 15 days after initial presentation.

We prescribed a combination of acetylsalicylic acid (ASA) 100 mg o.d. and ticagrelor 90 mg b.i.d., then followed by a life-long monotherapy with ASA 100 mg o.d. Heart failure therapy was initiated in accordance with current guidelines (sacubitril/valsartan 24 mg/26 mg b.i.d., bisoprolol 2.5 mg b.i.d., eplerenone 25 mg o.d., dapagliflozine 10 mg o.d.). We recommended screening for fibromuscular dysplasia (FMD) including imaging of cerebral arteries in an ambulatory setting, although coronary angiogram did not show the typical ‘string-of-beads’ sign for FMD, rendering it rather unlikely.

After 3-month follow-up, left ventricular systolic function had significantly improved (LVEF 55%) and while there was still scar evident, the extent of late gadolinium enhancement had significantly decreased on follow-up CMR (see Supplementary material online, *Figure A*, see Supplementary material online, *Video 7*).

## Discussion

Spontaneous coronary artery dissection is a serious and potentially life-threatening condition affecting primarily women and strongly associated with pregnancy. Management of P-SCAD does not greatly differ from non-pregnancy SCAD. Final diagnosis of P-SCAD is obtained through ICA.^1^ Special considerations in the cohort of pregnant patients should include minimization of radiation exposure, avoidance of teratogenic drugs, and evaluation of timing of delivery. Conservative management is preferred in stable patients with SCAD as most dissected segments usually heal spontaneously.^1^ Furthermore, a high incidence of iatrogenic coronary dissection within pregnant patients has been reported.^2^ A non-invasive approach may therefore be reasonable in patients with a low pre-test probability for AMI.

We opted for ICA initially in this case due to unstable angina and significantly elevated high-sensitive troponin T. Of note, IVUS was then crucial to confirm the diagnosis and to detect the extent of the coronary dissection during subsequent ICA. Intravascular imaging such as IVUS or optical coherence tomography is recommended to confirm guidewire position in the true lumen and show intramural haematoma as well as intimal tear.^3^ Additionally, IVUS may facilitate appropriate vessel sizing and optimize stent expansion. The use of intravascular imaging during initial ICA may therefore have led to diagnosis faster.

In general, for coronary artery dissection, it is recommended to limit PCI to severe cases with ongoing ischaemia, perform the minimum intervention necessary limiting the amount of stenting, and consider more widespread use of intravascular imaging to detect the extent of vessel wall disruption.^4^ Technical periprocedural risks of PCI include dissection or haematoma extension and side branch occlusion.^5,6^ Indications for revascularization in P-SCAD consist of total vessel occlusion, TIMI 0 flow, left main stem involvement, ongoing ischaemia, recurrent chest pain, haemodynamic instability, or ventricular arrhythmias.^1^ In our patient, revascularization was deemed appropriate due to persistent chest pain despite maximum antianginal medication and extensive scar on CMR. The stent was set within the proximal LAD. Nevertheless, propagation of the dissection into the diagonal branch occurred. In retrospect, conservative treatment may also have been a reasonable choice of treatment as our patient was haemodynamically stable at all times and did not present with total vessel occlusion or left main stem involvement. An alternative approach may also have been to apply a stent with bioresorbable vascular scaffold that allows for full recovery of the endothelial vessel function and has been previously described within SCAD patients.^7^

Adequate antiplatelet therapy is necessary after PCI and implantation of drug-eluting stents as reabsorption of the intramural haematoma increases the risk of stent malapposition and subsequent late stent thrombosis. Low-dose aspirin appears to be safe during pregnancy and is employed for prevention of pre-eclampsia as well.^8^ Current guidelines do not recommend the peripartal use of P2Y_12_ inhibitors due to limited data on their safety during pregnancy and nursing period.^9^ Dual antiplatelet therapy (DAPT) seems to be safe during pregnancy when applying low-dose aspirin and clopidogrel, however keeping in mind the increased bleeding-risk during pregnancy the duration of DAPT can be shortened.^8^ In contrast to atherosclerotic vessel disease, statin therapy has not proved to provide benefit and is hence not recommended for SCAD.^10^

Outcome for patients with P-SCAD overall is favourable. However, P-SCAD seems to predominantly involve the left main or left anterior descending coronary artery, resulting in considerable rates of reduced left ventricular function, life-threatening arrhythmias, or cardiogenic shock.^11^ The use of beta-blockers seems to be beneficial in SCAD patients, resulting in a reduced risk of recurrence.^12^

Due to the rarity of the disease, there is a paucity of data regarding the risk of pregnancy after SCAD. Generally, further pregnancy is not recommended. In case of subsequent pregnancy, close monitoring and individualized care with an interdisciplinary pregnancy heart team is recommended.

## Conclusion

In conclusion, P-SCAD is a rare but serious complication that should be taken into consideration when treating post-partum women with a new onset of chest pain. Suspicion of this entity and performing coronary angiography are key to an early diagnosis. If suspected, use of intravascular imaging is advisable to establish the diagnosis and guide treatment. Overall, outcome is favourable. We advocate for an interdisciplinary approach within a pregnancy heart team.

## Supplementary Material

ytae204_Supplementary_Data

## Data Availability

The datasets used and/or analysed during the current study are available from the corresponding author on reasonable request.

## Lead author biography

Dr Ailís Ceara Haney and Dr Deborah Siry are both doctors in training at the Department of Cardiology, Angiology and Pneumology at Heidelberg University Hospital. They are specializing in cardiac imaging.
