# Supplementary figures and images for: Spontaneous pregnancy-associated coronary artery dissection: a case report on diagnostic and therapeutic challenges
Source: Eur Heart J Case Rep. 2024 Apr 20;8(5):ytae204. doi: 10.1093/ehjcr/ytae204 (PMC11065351; doi:10.1093/ehjcr/ytae204)

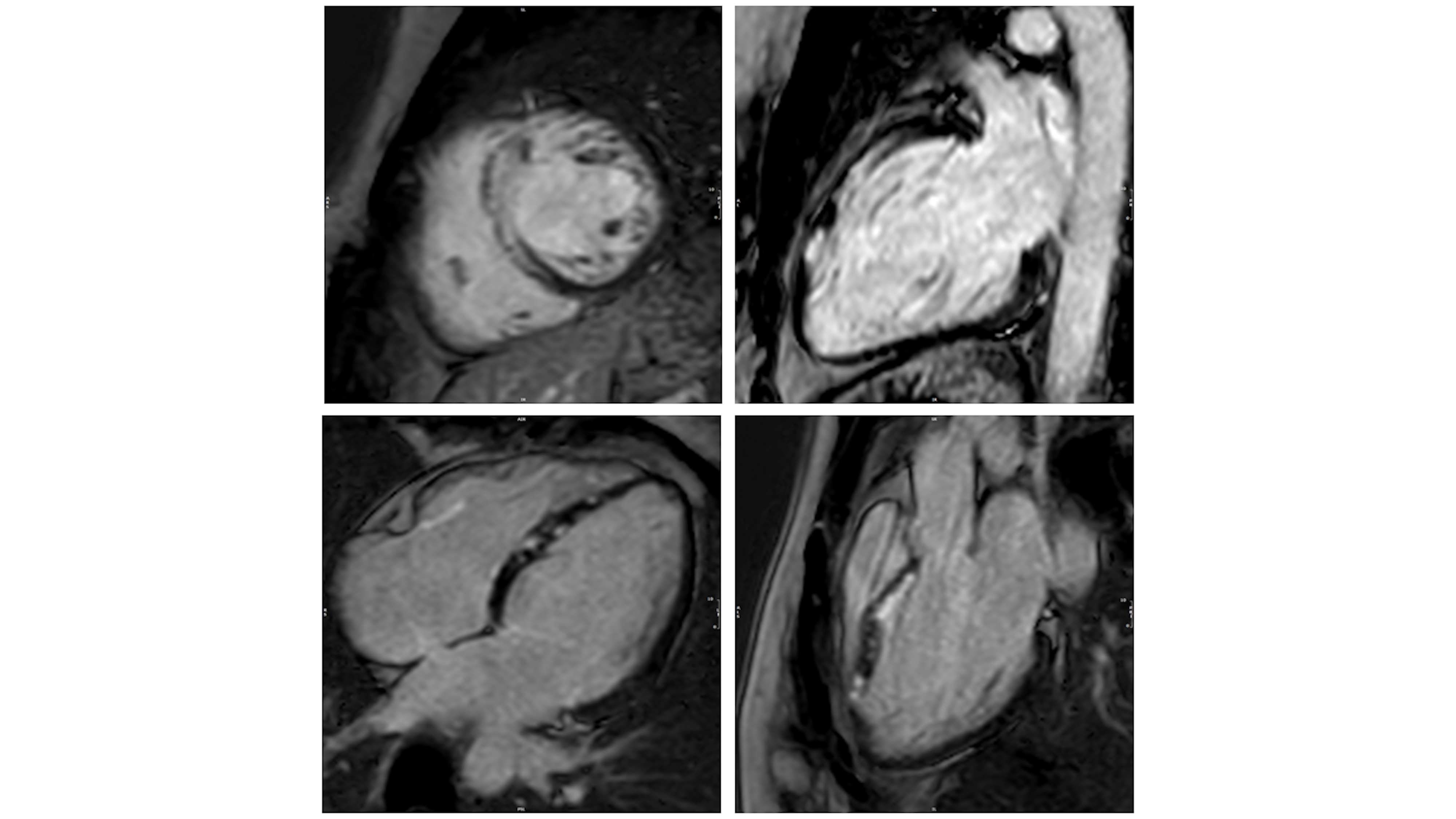

Supplement: ytae204_Supplementary_Data [file ytae204_supplementary_data.zip › Supplementary Figure A - LGE FU CMR.TIF]
